# IFN-γ alters the expression of diverse immunity related genes in a cell culture model designed to represent maturing neutrophils

**DOI:** 10.1371/journal.pone.0185956

**Published:** 2017-10-05

**Authors:** Michael A. Ellison, Christy M. Gearheart, Christopher C. Porter, Daniel R. Ambruso

**Affiliations:** 1 Department of Pediatrics, University of Colorado Denver, The Anschutz Medical Campus, Aurora, Colorado, United States of America; 2 Department of Pediatrics, Emory University School of Medicine, Atlanta, Georgia, United States of America; 3 Department of Pathology, University of Colorado Denver, The Anschutz Medical Campus, Aurora, Colorado, United States of America; 4 The Center for Cancer and Blood Disorders, Transfusion Services, Children's Hospital Colorado, Aurora, Colorado, United States of America; 5 Hematology/Oncology and Bone Marrow Transplantation Laboratories, Aurora, Colorado, United States of America; University of Thessaly Faculty of Medicine, GREECE

## Abstract

The cytokine interferon-γ (IFN-γ) is approved as a drug to treat chronic granulomatous disease (CGD) and osteopetrosis and is also used in hyperimmunoglobulin E syndromes. Patients with CGD have defects in proteins of the NOX2 NADPH oxidase system. This leads to reduced production of microbicidal ROS by PMNs and recurrent life threatening infections. The goal of this study was to better understand how IFN-γ might support phagocyte function in these diseases, and to obtain information that might expand potential uses for IFN-γ. Neutrophils mature in the bone marrow and then enter the blood where they quickly undergo apoptotic cell death with a half-life of only 5–10 hours. Therefore we reasoned that IFN-γ might exert its effects on neutrophils via prolonged exposure to cells undergoing maturation in the marrow rather than by its brief exposure to short-lived circulating cells. To explore this possibility we made use of PLB-985 cells, a myeloblast-like myeloid cell line that can be differentiated into a mature, neutrophil-like state by treatment with various agents including DMSO. In initial studies we investigated transcription and protein expression in PLB-985 cells undergoing maturation in the presence or absence of IFN-γ. We observed IFN-γ induced differences in expression of genes known to be involved in classical aspects of neutrophil function (transmigration, chemotaxis, phagocytosis, killing and pattern recognition) as well as genes involved in apoptosis and other mechanisms that regulating neutrophil number. We also observed differences for genes involved in the major histocompatibility complex I (MHCI) and MHCII systems whose involvement in neutrophil function is controversial and not well defined. Finally, we observed significant changes in expression of genes encoding guanylate binding proteins (Gbps) that are known to have roles in immunity but which have not as yet been linked to neutrophil function. We propose that changes in the expression within these classes of genes could help explain the immune supportive effects of IFN-γ. Next we explored if the effect of IFN-γ on expression of these genes is dependent on whether the cells are undergoing maturation; to do this we compared the effects of IFN-γ on cells cultured with and without DMSO. For a subset of genes the expression level changes caused by IFN-γ were much greater in maturing cells than non-maturing cells. These findings indicate that developmental changes associated with cell maturation can modulate the effects of IFN-γ but that this is gene specific. Since the effects of IFN-γ depend on whether cells are maturing, the gene expression changes observed in this study must be due to more than just prolonged application of IFN-γ and are instead the result of interplay between cell maturation and changes caused by the chemokine. This supports our hypothesis that the effects of IFN-γ on developing neutrophils in the bone marrow may be very different from its effects on mature cells in the blood. Collectively the findings in this study enhance our understanding of the effects of IFN-γ on maturing myeloid cells and indicate possible mechanisms by which this cytokine could support immune function.

## Introduction

The cytokine IFN-γ is approved as a drug to treat chronic granulomatous disease (CGD) and osteopetrosis and is also used in hyperimmunoglobulin E syndromes. These primary immunodeficiencies involve defects in neutrophils/polymorphonuclear cells (PMNs); patients with CGD have defects in proteins of the NADPH oxidase system which lead to reduced production of microbicidal ROS by PMNs [1], individuals with hyperimmunoglobulin E syndromes suffer from defective neutrophil chemotaxis resulting from decreased production of IFN-γ by their T lymphocytes [2] and patients with osteopetrosis have defective leukocytic-ROS production in addition to problems with bone resorption [3]. The goal of this study was to better understand how IFN-γ might support phagocyte function in these diseases, and to obtain information that might expand potential uses for IFN-γ. In particular we wanted to explore how CGD patients lacking essential protein components of the NADPH oxidase enzyme show clinical improvement while taking the drug as clearly demonstrated in a randomized, double blind, placebo-controlled study [4]. Although IFN-γ enhances production of the essential oxidase proteins in healthy individuals [5–8] this clearly cannot account for its beneficial effects in patients with a gene defect that leads to complete loss of a functional oxidase protein. Therefore characterization of other neutrophil-function-enhancing enhancing effects of IFN-γ is necessary to explain the clinical value of the drug.

Neutrophils mature in the bone marrow and then enter the blood where they quickly undergo apoptotic cell death; a range of human studies have estimated the neutrophil half-life at only 5–10 hours and a single report estimating their half-life at 3.75 days has been challenged as primarily reflecting the time course of their maturation in the marrow [9]. Therefore we reasoned that IFN-γ might exert its effects on neutrophils via prolonged exposure to cells undergoing maturation toward a terminally differentiated state in the marrow rather than by its brief exposure to short-lived circulating cells. To explore this possibility we made use of PLB-985 cells which are a myeloblast-like myeloid cell line that can be differentiated into a mature, neutrophil-like state by treatment with various agents including DMSO [10;11].

In initial studies we investigated transcription and protein expression in PLB-985 cells undergoing maturation in the presence or absence of IFN-γ. We observed IFN-γ induced differences in expression of genes known to be involved in classical aspects of neutrophil function (transmigration, chemotaxis, phagocytosis, killing and pattern recognition) as well as genes involved in apoptosis and other mechanisms that regulating neutrophil number. We also observed differences for genes involved in the major histocompatibility complex I (MHCI) and MHCII systems whose involvement in neutrophil function is controversial and not well defined. Finally, we described significant changes in expression of genes encoding guanylate binding proteins (Gbps) that are known to have roles in immunity but which have not as yet been linked to neutrophil function. We propose that changes in the expression within these classes of genes could help explain the immune supportive effects of IFN-γ.

Next we explored if the effect of IFN-γ on expression of these genes is dependent on whether the cells are undergoing maturation; to do this we compared the effects of IFN-γ on cells cultured with and without DMSO. For a subset of genes the expression level changes caused by IFN-γ were much greater in maturing cells than non-maturing cells. These findings indicate that developmental changes associated with cell maturation can modulate the effects of IFN-γ but that this is gene specific. Since the effects of IFN-γ are dependent on whether cells are maturing, the gene expression changes observed in this study must be due to more than just prolonged application of IFN-γ and are instead the result of interplay between cell maturation and changes caused by the chemokine. This supports our hypothesis that the effects of IFN-γ on developing neutrophils in the bone marrow may be very different from its effects on mature cells in the blood.

Collectively the findings in this study enhance our understanding of the effects of IFN-γ on maturing myeloid cells and indicate possible mechanisms by which this cytokine could support immune function.

## Methods

### Cell culture, maturation and treatment with IFN-γ

This was essentially as described previously [12]. Briefly, PLB-985 cells from the German Collection of Microorganisms and Cell Cultures (DSMZ) were grown at 37°C in suspension in RPMI 1640 medium containing 2mM L-glutamine (Gibco), 10% bovine serum (Atlanta Biologicals) and 100 units/mL penicillin plus 100 mg/mL streptomycin (Sigma Life Science). The cultures were maintained in a humidified, 5% CO_2_ atmosphere. The cells were split to 0.2X106/ml every 3 to 4 days. The cells would double in slightly longer than 24 hours and reach about 3.0X106/ml before reductions in growth rate were seen.

For DMSO-induced cell-maturation, PLB-985 cells (1–1.5X10^6^/ml) in log phase growth were pelleted (250XG, 5min) and re-suspended to 0.4X10^6^/ml in the standard media plus 1.3% (vol:vol) DMSO (Sigma-Aldrich). This was done with or without including 30ng/ml of IFN-γ (Horizon Pharma Ireland Ltd) to model maturation in the presence or absence of IFN-γ. To determine the effects of IFN-γ on non-maturing cells, the cells were re-suspended in either the standard media alone (without DMSO) of the standard media without DMSO plus 30ng/ml IFN-γ. In both cases, after 72 hours the cells were harvested and used for preparation of RNA or cell lysates as described below.

To determine the effects of IFN-γ applied to already mature PLB-985 cells, cells differentiated for 72 hours in 1.3% DMSO were pelleted and resuspended in fresh media containing, or lacking, 30ng/ml IFN-γ for a further 3 hours. RNA preparation and cell lysis was then done as described below.

In total four independent experiments were performed for each of the experimental conditions (+/- IFN-γ in both maturing and non-maturing cells and +/- IFN-γ applied to already mature cells) and RNA from each experiment was independently used in microarray studies (see below).

### mRNA measurement by microarray

PLB-985 cells were treated as described under Cell Culture, Maturation and Treatment with IFN-γ. Aliquots containing one million cells were pelleted and stored at -70°C until use. Total RNA was extracted using the RNeasy Mini Kit (Qiagen).

All further microarray work was done at the University of Colorado Genomic and Microarray Core Facility. Preparation of labelled cDNA, hybridization to HuGene2.0ST microarrays (Affymetrix), microarray washing and staining (using a GeneChip Fluidics Station 450) and microarray scanning (using a GeneChip Scanner 3000) were done as described in the appropriate Affymetrix manuals (GeneChip WT PLUS Reagent Kit, AffymetrixGeneChip Fluidics Station 450 User's Guide, the GeneChip Expression Wash, Stain, and Scan User Manual for Cartridge Arrays (PN 702731), and the Affymetrix GeneChip Command Console User Manual).

Data collected from microarray analysis were preprocessed using the Affymetrix Expression Console (Build 1.3.1.187) to be RMA-normalized then log2 transformed to minimize technical variation among samples across multiple arrays [13]. Relative expression levels were then compared and one-way unpaired ANOVA analysis was used to determine p-values to assign significance for the differences in gene expression levels for DMSO plus IFN-γ treated cells versus DMSO treated cells and also for IFN-γ treated cells versus untreated (maintained in media only) cells.

### Western blotting

PLB-985 cells were pelleted at 250XG for 5min, were resuspended in 1ml of ice cold KRP-d (12.5 mM Na_2_HPO_4_, 3 mM NaH_2_ PO_4_, 4.8 mM KCl, 120 mM NaCl, 1.3 mM CaCl_2_, 1.2 mM MgSO_4_, 0.2% dextrose, pH 7.3–7.4) and were re-centrifuged with the resulting pellets being stored stored at -70°C until use. After thawing, cells were resuspended in 1ml of lysis buffer (150mM NaCl, 50mM Tris-HCl, 50mM NaF, 5mM EDTA, 0.5% (weight:volume) sodium deoxycholate, 0.1% (weight:volume) sodium dodecyl sulfate (SDS), 1% (volume:volume), Triton X-100, pH7.6) containing a protease inhibitor mix obtained by dissolving EDTA free-cOmplete Ultra Tablets (Roche) according to the manufacturer’s instructions. Cells were the disrupted by sonication for 30 seconds at 30% power in a model W-220F cell disruptor (Heat Systems-Ultrasonics Inc.). The total protein concentration in the cell lysates was determined by BCA Protein Assay Kit (Pierce) and the cell lysates were stored at -70°C until use.

Proteins from cell lysates were resolved by SDS-PAGE. The amount of total protein loaded per well varied by detected protein as follows; 10μg for GAPDH, 20μg for TLR4, NLRP3, AIM2, GBP5, HLA-DRA, CD74, HLA-A, FCGR1A, ITGA4, ITGB7, MPO and CASP7 and 50μg for CASP1, LY96 and FAS. Proteins were transferred (400 mA, 1 hour) in a Mighty Small Transphor transfer tank (GE Life Science) to activated polyvinylidene flouride membranes (LY96) or nitrocellulose membranes (for all other proteins) in buffer containing 20% (volume:volume) methanol, 0.025 M glycine, and 0.0015% (volume:volume) ethanolamine.

For detection of proteins, membranes were exposed to antibodies diluted in 10% (weight:volume) non-fat dry milk (NFDM) in TBS-T (20mM Tris, 137mM NaCl, 0.1% (volume:volume) Tween-20, pH 7.6). The following primary antibodies were applied overnight at 4°C and a 1:500 (HLA-DRA, HLA-A, TRAIL and FAS) or 1:1000 dilution (all other proteins): mouse anti-GAPDH (Santa Cruz Biotechnology sc-47724)**;** rabbit anti-TLR4 (Novus Biological NBP2-24538); rabbit anti-NLRP3 (Cell Signaling Technology (D2P5E) #13158); rabbit anti-CASP1 (Cell Signaling Technology #2225); rabbit anti-AIM2 (Origene TA314913); rabbit anti-GBP5 (Origene TA502286); rabbit anti-LY96 (Novus Biological NB100-56655); rabbit anti-HLA-DRA (Origene TA322280); rabbit anti-CD74 (Santa Cruz Biotechnology sc-20082); goat anti-HLA-A (Santa Cruz Biotechnology sc-23446); mouse anti-FCGR1A (Origene TA506343); rabbit anti-ITGA4 (Cell Signaling (D2E1) XP #8440); rabbit anti-ITGB7 (Origene TA307663); rabbit anti-MPO (Cell Signaling Technology (E1E7I) XP #14569); rabbit anti-CASP7 (Cell Signaling Technology #9492); rabbit anti-FAS (Cell Signaling Technology (C18C12) #4233) and; rabbit anti-TRAIL (Cell Signaling Technology (C92B9) #3219. The secondary antibodies, at a 1:1000 dilution in 10% (weight:volume) NFDM in TBS-T were peroxidase linked anti-rabbit IgG or anti-mouse IgG (NA9340V and NA931V respectively from GE Healthcare) or peroxidase linked anti-goat IgG (605-703-125 from Rockland Immunochemicals Inc.) as appropriate and were applied for 1hr at room temperature. Immune complexes were then detected, according to the manufacturer’s instructions, with Luminata Forte Western HRP Substrate (Millipore) for HLA-DRA, HLA-A, TRAIL and FAS or Enhanced Chemiluminescence Western Blotting Detection Reagents (GE Healthcare) for all other proteins. All other standard chemicals for western blotting were from Sigma-Aldrich, Acros or Fisher.

## Results

### Several groups of genes with either well characterized or less well characterized but compelling ties to neutrophil function are upregulated by IFN-γ treatment in maturing PLB-985 cells

Using microarrays we compared mRNA levels in cells matured using DMSO in the presence of IFN-γ, (DMSO plus IFN-γ) and cells matured using DMSO in the absence of IFN-γ (DMSO). In Tables 1–5 we report the fold increase in mRNA for the DMSO plus IFN-γ treated cells relative to the DMSO treated cells. A number of genes (Tables 1–5) with known or potential roles in neutrophil function were found to be modulated by IFN-γ treatment. They are outlined below and are described more fully in the Discussion.

**Table 1:** Proteins involved in basic neutrophil functions.

Gene	Common Protein Name(s)	Fold change for DMSO plus IFN-γ treatment versus DMSO treatment	ANOVA p-value
Proteins Involved in Neutrophil Transmigration			
SELPLG	P Selectin Glycoprotein Ligand 1	1.21	0.000233
ITGAM	Integrin Subunit αM/CD11b	1.32	0.00902
ITGAL	Integrin Subunit αL/CD11a	1.84	0.000057
ITGAX	Integrin Subunit αX/CD11c	1.33	0.044166
ITGA4	Integrin Subunit α4	2.13	0.000031
ITGB7	Integrin Subunit β7	6.03	1.91E-07
CEACAM1	Receptor CD66a	10.42	0.000003
SELL	L-selectin	3.31	0.000357
SYK	Spleen Associated Tyrosine Kinase	-1.4	0.000142
FGR	Fgr/FGR Proto-Oncogene, Src Family Tyrosine Kinase	-1.72	0.000025
LYN	Lyn/LYN Proto-Oncogene, Src Family Tyrosine Kinase	1.47	0.006379
Proteins Involved in Chemotaxis			
FPR1	Formyl Peptide Receptor 1	1.66	0.000256
FPR2	Formyl Peptide Receptor 2	1.48	0.001889
C3AR1	Complement Component 3a Receptor 1	1.72	0.00363
Proteins Involved in Phagocytosis			
FCGR1A	Fc Gamma Receptor Ia/FcγRIA	39.77	6.62E-07
FCGR1B	Fc Gamma Receptor Ib/FcγRIB	5.33	0.000007
FCGR2A	Fcγ receptor IIa/FcγRIIA	1.81	0.000032
FCGR2B	Fcγ receptor IIb/FcγRIIB	2.12	0.047517
FCER1G	FcRγ	1.48	0.000019
Proteins involved in killing of pathogens			
CYBB*	gp91-Phox	1.19	0.010271
NCF1*	p47-Phox	1.51	0.025401
LYZ	Lysozyme	1.3	0.005024
MPO	Myeloproxidase	-22.24	4.90E-07

^a^This data was originally reported in Ellison MA et. al [12]

**Table 2:** Proteins involved in neutrophil clearance and homeostasis.

Gene	Common Protein Name(s)	Fold change for DMSO plus IFN-γ treatment versus DMSO treatment	ANOVA p-value
CASP10	Caspase 10	3.05	0.000006
CASP7	Caspase 7	2.3	0.000016
CASP3	Caspase 3	1.73	0.000026
CAPN3	Calpain 3	2.04	0.019004
CAPNS2	Calpain Small Subunit 2	1.64	0.006169
CAST	Calpstatin	1.39	0.000024
FAS	Fas Cell Surface Death Receptor	5.35	0.000002
TNFRSF1A	Tumor Necrosis Factor- α Receptor	1.31	0.001167
TNFSF10	TNF-Related Apoptosis-Inducing Ligand/TRAIL	10.59	1.18E-08
BAX	BCL2 Associated X Protein	1.29	0.004979
BAK1	BCL2 Antagonist/Killer 1	2.05	0.000007
BCL2L11	BCL2 Like 11	1.38	0.019261
BMF	BCL2 Modifying Factor	1.24	0.002022
BCL2L1	BCL2 Like 1	1.62	0.002288
MCL1	Myeloid Cell Leukemia 1	1.69	0.000514
BCL2A1	BCL2 Related Protein A1	1.56	0.00111
APOL6	Apolipoprotein L6	6.6	1.11E-07
XIAP	X-Linked Inhibitor Of Apoptosis	1.55	0.01186
XAF1	XIAP Associated Factor 1	23.45	4.71E-07
CXCR4	C-X-C Motif Chemokine Receptor 4	-1.98	0.000137

**Table 3:** Innate immune receptors.

Gene	Common Protein Name(s)	Fold change for DMSO plus IFN-γ treatment versus DMSO treatment	ANOVA p-value
Toll Like Receptors			
TLR4	Toll Like Receptor 4	2.14	0.000095
LY96	Lymphocyte Antigen 96	5.96	0.000844
TLR8	Toll Like Receptor 8	5.5	2.26E-07
TLR1	Toll Like Receptor 1	2.1	0.000152
TLR2	Toll Like Receptor 2	-1.5	0.020288
TLR3	Toll Like Receptor 3	1.44	0.012239
TLR9	Toll Like Receptor 9	-1.28	0.010429
MYD88	Myeloid Differentiation Primary Response 88	1.48	0.000243
RIPK1	Receptor-Interacting Protein 1	1.28	0.000001
RIPK2	Receptor-Interacting Protein 2	2.6	0.000913
TANK	TRAF Family Member Associated NFKB Activator	1.48	0.002157
TRAF6	Interleukin-1 Signal Transducer	1.31	0.000461
IRAK3	IRAK-M	-1.59	0.000721
Nod-Like Receptors, Inflammasome Components and AIM2			
NOD1	Nucleotide Binding Oligomerization Domain Containing 1	1.38	0.003572
NLRP3	NLR Family, Pyrin Domain Containing 3	1.78	0.000068
CASP1	Caspase 1	5	5.47E-07
PYCARD	PYD And CARD Domain Containing	1.37	0.000337
AIM2	Absent in Myeloma 2	24.56	0.000016
C-type lectins			
CLEC7A	C-Type Lectin Domain Family 7 Member A	1.6	0.001515
CLEC12A	C-Type Lectin Domain Family 12 Member A	-2.23	0.000048
CLEC4E	C-Type Lectin Domain Family 4 Member E	-1.43	0.028254
CLEC4D	C-Type Lectin Domain Family 4 Member D	-1.55	0.011814
CLEC2B	C-Type Lectin Domain Family 2 Member B	8.15	8.55E-07
CLEC5A	C-Type Lectin Domain Family 5 Member A	-2.4	0.018912

**Table 4:** Guanylate binding proteins.

Gene	Common Protein Name(s)	Fold change for DMSO plus IFN-γ treatment versus DMSO treatment	ANOVA p-value
GBP5	Guanylate Binding Protein 5	337.28	1.97E-09
GBP4	Guanylate Binding Protein 4	248.98	3.31E-09
GBP2	Guanylate Binding Protein 2	213.09	8.41E-09
GBP1	Guanylate Binding Protein 1	188.25	9.86E-07
GBP3	Guanylate Binding Protein 3	22.21	2.33E-08
GBP6	Guanylate Binding Protein 6	2.43	0.003274

**Table 5:** Proteins involved or potentially involved in antigen presentation by MHCII and MHCI.

Gene		Common Protein Name(s)	Fold change for DMSO plus IFN-γ treatment versus DMSO treatment	ANOVA p-value
Proteins Involved or Potentially Involved in Antigen Presentation by MHCII				
CIITA	Class II, Major Histocompatibility Complex, Transactivator		6.35	0.000019
RFX5	Regulatory Factor X5		1.47	0.00167
HLA-DRA	MHC, Class II, DR α		74.97	1.64E-07
HLA-DPA1	MHC, Class II, DP α 1		4.79	0.000027
HLA-DPB1	MHC, Class II, DP β 1		3.06	0.000052
CD74	Invariant Chain/iI/CD74 molecule		20.84	0.000005
HLA-DMA	MHC, Class II, DM α		2.98	0.000059
HLA-DMB	MHC, Class II, DM β		2.3	0.000002
CD86	CD86 antigen		1.5	0.000185
CD40	CD40 antigen		11.95	0.000004
PDCD1LG2	CD273 antigen		11.63	0.000007
CD274	CD274 antigen		35.96	3.44E-07
CTSB	Cathepsin B		1.49	0.000812
CTSL1	Cathepsin L1		1.47	0.024585
CTSS	Cathepsin S		2.97	3.69E-07
CTSO	Cathepsin O		9.25	0.000004
CTSZ	Cathepsin Z		1.78	0.000096
CTSA	Cathepsin A		1.64	0.000184
Proteins Involved in Antigen Presentation by MHCI				
B2M		Beta-2-Microglobulin	1.54	0.000069
HLA-A		MHC, Class I, A	2.36	0.000477
HLA-B		MHC, Class I, B	2.55	0.000587
HLA-C		MHC, Class I, C	2.01	0.000006
HLA-E		MHC, Class I, E	2.73	3.05E-09
HLA-F		MHC, Class I, F	1.62	0.000345
HLA-G		MHC, Class I, G	2.21	0.000009
TAP1		Peptide Transporter Involved In Antigen Processing 1	20.86	1.57E-09
TAP2		Peptide Transporter Involved In Antigen Processing 2	11.88	1.60E-08
TAPBP		TAP Binding Protein (Tapasin)	2.59	0.000004
PDIA3		Protein Disulfide Isomerase Family A Member 3	1.75	0.000288
CALR		Calreticulin	-1.24	0.002063

We first examined whether treatment with IFN-γ caused changes in the levels of mRNAs for genes that are involved in basic neutrophil functions, i.e., transmigration from the circulation into the tissue, chemotaxis to a site of infection and phagocytosis and killing of microorganisms. A number of genes involved in these processes were found to have significantly altered transcription after maturation in the presence of IFN-γ (Table 1). Because genes involved in neutrophil clearance and homeostasis are important for regulating neutrophil numbers in response to infection and for the orderly removal of neutrophils which have ingested pathogens we looked for changes in genes involved in these processes and again found a number of genes that were significantly up or down regulated (Table 2).

Neutrophils express many innate immune receptors which bind to molecules that are pathogen specific and are distinguishable from host molecules (pathogen associated molecular patterns or PAMPS). Because binding of these receptors to their corresponding ligands triggers anti-microbicidal and pro-inflammatory changes we looked for IFN-γ mediated changes in mRNAs encoding innate immune receptors or associated factors and found that a number of relevant genes were effected (Table 3).

Next we examined which genes displayed the highest level of upregulation in PLB-985 cells matured in the presence of IFN-γ relative to those matured in the absence of IFN-γ We found that several guanylate binding proteins (GBPs) showed the highest levels of upregulation of any gene. Although roles for these proteins are not characterized in neutrophils, many of them have well documented anti-microbicidal roles in other immune cells. Because GBPs might function in similar capacities in neutrophils, their upregulation by IFN-γ might help explain the immunity enhancing properties of IFN-γ. Therefore we documented the upregulation of all GBPs modulated by IFN-γ in this study (Table 4).

We also found that one of the transcripts most upregulated by IFN-γ corresponded to a gene encoding a subunit of one of the MHCII molecules (HLA-DRA). As outlined in the Discussion, the MHCI and II systems are involved in antigen presentation and activation of lymphocytes within adaptive immunity. Despite the fact that neutrophils are not generally considered to be antigen presenting cells, emerging evidence suggest that they may be involved in antigen presentation and activation of T-lymphocytes. Thus we examined whether the IFN-γ treatment upregulated genes involved in the MHCI and MHCII systems and the findings are shown in Table 5.

**Fig 1:**
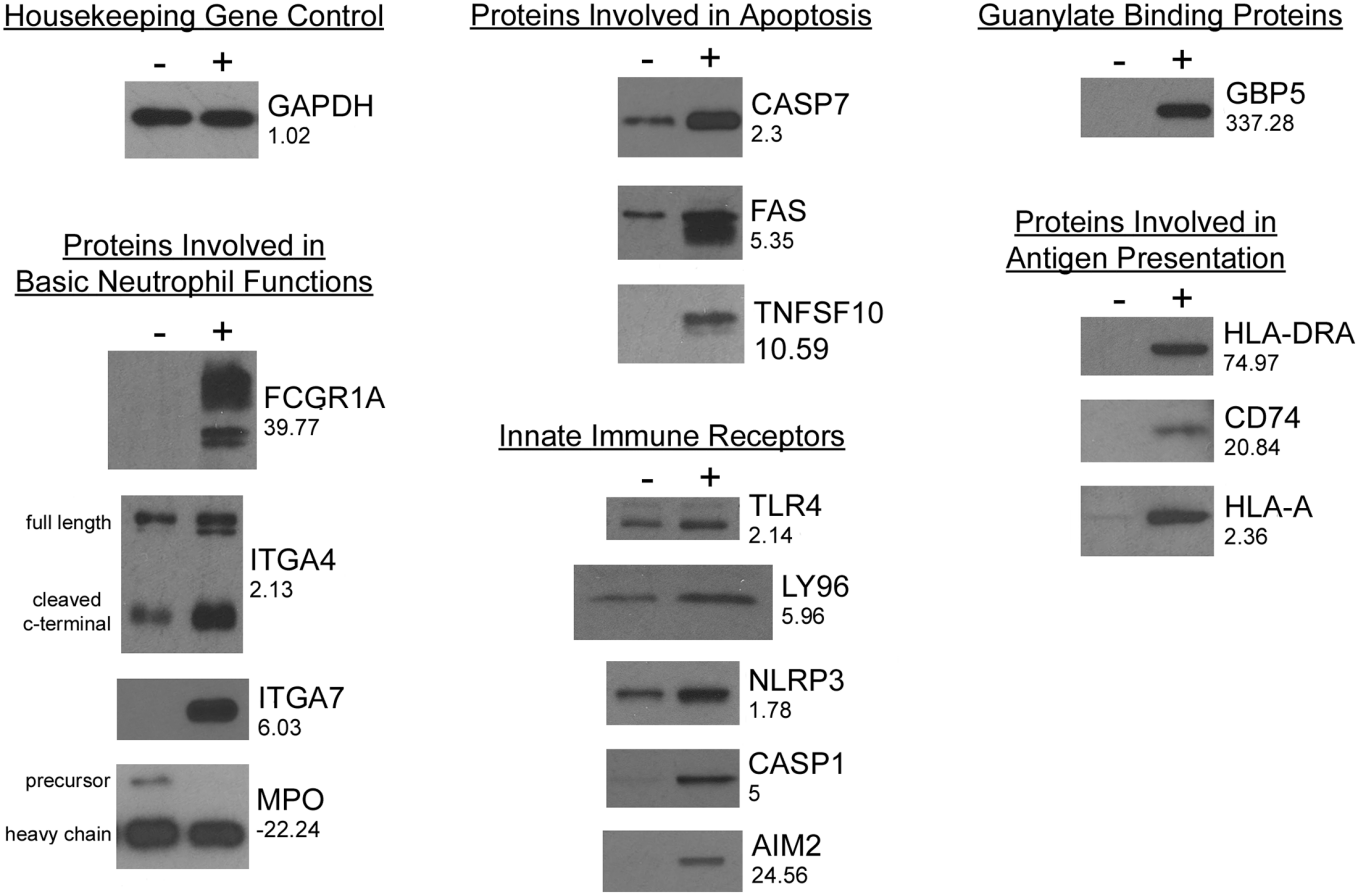
Western blots to correlate changes in mRNA levels with corresponding protein levels. The levels of select proteins in Tables 1–5 were investigated by Western Blot. Lanes containing cell lysate from untreated cell are on the left (-) and lysates from IFN-γ treated cells are on the right (+). The blots are organized according to the roles of the proteins in the same way as in Tables 1–5. The proteins are identified by the corresponding gene names. The housekeeping enzyme glyceraldehyde 3-phosphate dehydrogenase (GAPDH) was blotted as a control that is not altered by IFN-γ treatment. Except for GAPDH, the numbers under each gene name are the fold changes in mRNAs as in Tables 1–5. For GAPDH the 1.02 fold change was not significant (ANOVA p-value = 0.444976).

To confirm that the observed changes in microarray output reflect changes in mRNA levels and consequent changes in protein expression we carried out western blots on cell lysates from PLB-985 cells matured in the presence and absence of IFN-γ. These results are shown in Fig 1 and indicate that protein level changes match the changes in microarray signal quite well. However, in the case of HLA-A, upregulation of the protein appears to be greater than the 2.36 fold upregulation of the corresponding mRNA suggesting that additional factors such as increased protein or mRNA stability or enhanced translation might also be caused by IFN-γ with respect to HLA-A. Additionally, in the case of myeloperoxidase, although myeloperoxidase -precursor protein was down regulated dramatically consistent with the large reduction in its mRNA, the level of heavy chain protein produced from the precursor was down less (Fig 1).

### Certain genes show much larger IFN-γ-induced changes in expression in maturing cells than non-maturing cells

**Fig 2:**
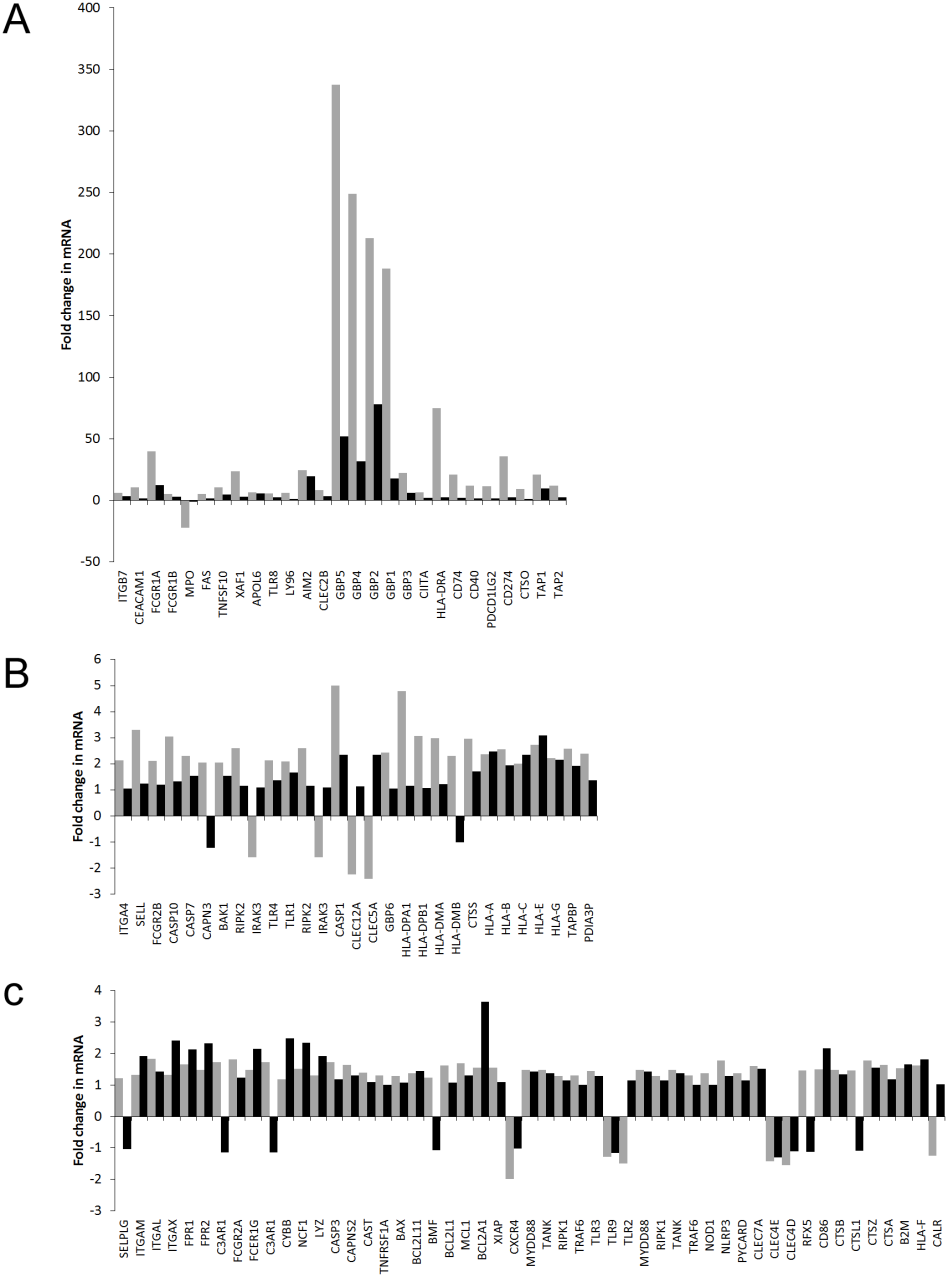
Comparison of the effect of IFN-γ in maturing cells and non-maturing PLB-985 cells. For the immune related genes in Tables 1–5, the fold change in mRNA levels for DMSO plus IFN-γ treated cells versus DMSO treated cells (grey bars) were compared to the fold changes for IFN-γ treated cells versus untreated cells (black bars). The DMSO plus IFN-γ versus DMSO fold change models the effect of IFN-γ on maturing cells whereas the IFN-γ versus untreated fold change represents its effect on non-maturing cells. Graphs A, B and C show the results for those genes where the DMSO plus IFN-γ versus DMSO fold change (increase or decrease) was greater than 5 fold, 2 to 5 fold or less than 2 fold, respectively.

We explored whether the effect of IFN-γ on expression of the immune related genes in Tables 1–5 is dependent on whether the cells are undergoing maturation. In our model system, the fold changes in mRNA levels for DMSO plus IFN-γ treated cells versus DMSO treated cells represent the effect of IFN-γ on gene expression in maturing cells. Similarly, the effect of IFN-γ in the absence of maturation is given by the fold changes for IFN-γ treated cells versus untreated cells. Thus, to explore the effect of IFN-γ on maturing versus non-maturing cells we compared the fold changes in mRNA levels found in DMSO plus IFN-γ treated cells versus DMSO treated cells to the fold changes for IFN-γ treated cells versus untreated cells (Fig 2).

We found that IFN-γ application to maturing cells often caused much larger changes in gene expression than application to non-maturing cells. However this effect was restricted to those genes that were most strongly affected by IFN-γ application during maturation. For those genes where IFN-γ applied during maturation caused a greater than 5 fold increase in expression, all corresponding fold changes induced by IFN-γ applied to non-maturing cells were much smaller (Fig 2A). This difference was less obvious when the expression change induced by IFN-γ during maturation was between 2 and 5 fold (Fig 2B) and even less clear at less than 2 fold (Fig 2C).

Examination of genome wide changes in transcription also supported the idea that IFN-γ has a more dramatic effect when applied to maturing cells. Comparison of the transcription changes when IFN-γ was applied to maturing cells (INF-γ plus DMSO versus DMSO) indicated that there was a significant (ANOVA p-value<0.05) increase in 2928 genes and a significant decrease in 3553 genes. For IFN-γ application to non-maturing cells (IFN-γ treated cells versus untreated cells) however there were fewer increases (1958 genes) and decreases (1763 genes)

### IFN-γ-induced alterations of gene expression in maturing or already mature PLB-985 correlate only for certain genes

PLB-985 cells matured for 72 hours in DMSO are terminally differentiated and short lived (about 75% are permeable to trypan blue after a further 24 hours in culture). Therefore they cannot be treated with IFN-γ for prolonged times. Nevertheless, the distinct effects of IFN-γ applied during DMSO mediated maturation might be the result of its effect on cells that are approaching full differentiation; on the other hand, they might be due to the presence of the drug throughout maturation including during the early stages when the cells are more immature. To investigate these possibilities we either applied, or did not apply, IFN-γ for 3 hours to PLB-985 cells that had already been matured with DMSO for 72 hours. Using microarrays we than compared mRNA levels in cells under these two conditions. For each gene in Tables 1–5, the fold change in mRNA in mature cells treated for 3 hours relative to mature cells that were untreated are shown in S1 Table. The corresponding fold changes when maturing cells were treated with IFN-γ relative to when they were untreated (from Tables 1–5) are also shown for comparison.

As can be seen in S1 Table, for some genes, (e.g. for CASP7) 3 hours of application of IFN-γ to mature cells caused changes in expression that were similar to the changes seen when IFN-γ was applied for 72 hours to maturing cells. For these genes it thus appears that the effects of IFN-γ are elicited by its effect on cells approaching or at full differentiation. Two clear examples of this are shown in Table 6, FCGR1A was upregulated 39.77 fold when IFN-γ was applied for 72 hours to maturing cells and was also up 22.57 fold after only 3 hours of application to already mature PLB-985 cells. Similarly, APOL6 was upregulated 6.6 fold when IFN-γ was applied during maturation and was up slightly more (7.65 fold) when applied to already mature cells.

**Table 6:** Gene dependent differences in expression changes induced by IFN-γ applied to already mature or maturing cells.

Gene	Fold change for mature cells treated with IFN-γ versus untreated mature cells	Fold change for DMSO plus IFN-γ treatment versus DMSO treatment
FCGRIA	39.77	22.57
APOL6	6.6	7.65
MPO	-22.24	-1.05^a^
XAF	23.45	1.12^a^
HLA-DRA	74.97	1.32^a^
GBP5	337.28	13.36

^a^ Non-significant changes (p>0.05)

FCGRIA and APOL6 are examples of genes that were strongly upregulated when IFN-γ was applied to already mature cells for 3 hours. In contrast, the other genes in Table 6 were strongly modulated by IFN-γ when cells were treated during maturation but their expression was much less altered when IFN-γ was applied to already mature cells.

In contrast, some genes showed very little or no changes in expression when applied to already mature cells for 3 hours and large up or down regulation when applied to maturing cells for 3 days e.g. CAPN3 (see S1 Table). Striking examples of this are shown in Table 6. MPO, XAF and HLA-DRA expression were down 22.24, up 23.45 and up 74.97 fold when applied during maturation but were unchanged (p>0.05) when applied for 3 hours to mature cells. Similarly, GBP5 expression was up 337.28 fold when IFN-γ was applied during maturation but was only up 13.36 fold when applied to already matured cells.

Thus it appears that there are gene specific differences in whether the effects of IFN-γ are caused by its application to mature cells or by its presence during maturation.

## Discussion

As shown in Tables 1–3, the levels of many mRNAs encoding proteins with well-established roles in neutrophil function were different in PLB-985 cells matured in the presence of IFN-γ relative to cells matured in its absence. These genes include those which are involved in the basic steps of the neutrophil life cycle, those involved in neutrophil homeostasis and clearance and those which encode innate immune receptors. Furthermore, as shown in Tables 4 and 5, we identified a number of transcripts that were significantly altered by IFN-γ and which are immunity related but for which a role in PMNs is unreported or is subject to conflicting experimental data (guanylate binding proteins and proteins of the MHCI and MHCII systems). As shown in Fig 1, the corresponding proteins for a selection of these mRNAs were examined by Western Blot and protein level changes corresponded well with mRNA changes.

The alteration of the expression of these immunity related genes by IFN-γ could thus account, at least in part, for the immune-supportive role of IFN-γ in patients treated with this chemokine. These proteins and ways in which their modulation by IFN-γ might potentially alter neutrophil function are explored in more detail below. In this section proteins are identified by a common name or names followed by the corresponding gene name in parenthesis.

### Proteins involved in basic neutrophil functions that are central to microbicidal activity

#### Proteins involved in neutrophil transmigration

To reach a site of infection and inflammation PMNs must leave the circulation and transmigrate into the surrounding tissue [14]. This occurs at endothelial sites which have been activated by molecular signals from nearby infection or tissue damage. First, PMNs attach weakly to the endothelium and roll along its surface but then tight PMN-endothelial binding arrests rolling and leads to firm adherence. Next PMNs crawl over the endothelial surface and, after reaching a suitable site, they move between endothelial cells into the underlying tissue. These different phases of attachment are mediated by sequential associations between specific cell surface molecules on PMNs and endothelium. In this study we find that the mRNAs of several proteins implicated in this process are upregulated in PLB-985 cells matured in the presence of IFN-γ relative to cells matured in its absence (Table 1). Collectively these observations suggest a possible mechanism by which INF-γ might enhance PMN transmigration from the blood stream.

As shown in Table 1, the mRNA encoding P-selectin glycoprotein ligand 1/PSGL-1 (SELPLG) was upregulated 1.21 fold in PLB-985 cells following INF-γ treatment (Table 1). This protein occurs on the surface of PMNs and mediates rolling by interacting with P or E-selectin on endothelium [14]. Based on our PLB-985 model we thus suggest that upregulation of this protein by IFN-γ might thus enhance neutrophil-endothelium binding and subsequent transmigration.

We also observed upregulation of certain integrins on PLB-985 cells differentiated in the presence of IFN-γ. Integrins are a family of transmembrane proteins consisting of non-covalently linked α and β subunits [15–17]. Neutrophils mainly express β2 containing integrins including β2αM, β2αL and β2αX. Although β2 integrin was not upregulated by IFN-γ in differentiating PLB-985 cells, αM/CD11b (ITGAM), αL/Cd11a (ITGAL) and αX/CD11c (ITGAX) were (Table 1). β2αM, β2αL and β2αX on the surfaces of PMNs interact with proteins belonging to the intercellular adhesion molecule (ICAM) family on the surfaces of activated endothelial cells and ICAM-integrin binding arrests rolling, promotes tight PMN-endothelium binding and facilitates crawling. β2αM and β2αL have also been implicated in specific phases of transmigration through the endothelium [18]. Thus, based on the PLB-985 cell model used in this study, up regulation of integrins is another potential mechanism by which PMN transmigration could be enhanced by IFN-γ. Strikingly, the IFN-γ induced upregulation of ITGAM which we noted is consistent with a previous study looking at the protective effects of INF-γ on stored neutrophils [19]. In this work it was found that incubation with IFN-γ slowed the decline of the oxidative burst, bactericidal activity and adherence in stored neutrophils. This protection correlated with a decreased reductions in ITGAM mRNA and cell surface expression over time. Thus, for ITGAM there is a consistent effect of IFN-γ in neutrophils and our model system.

Although β2 containing integrins are thought to be required for most neutrophil transmigration, in the lung β2-integrin-independent PMN emigration has been described [20]. It has been shown that α4β1 and α5β1 integrins function as major alternatives to β2-containing integrins in mediating PMN migration to alveoli [21]. Furthermore α4β7 integrin mediates attachment of blood borne lymphocytes to lamina propria venules in situ and β4 containing integrins initiate lose lymphocyte contact and rolling in vitro [22]. These interactions appear to be due to binding of α4β7 integrins to the endothelial ligands MAdCAM-1 and VCAM-1 [22] and similar protein-protein associations could potentially be important in neutrophils. In our study mRNAs encoding both α4 (ITGA4) and β7 (ITGB7) integrins were upregulated in the PLB-985 model (Table 1) suggesting that IFN-γ treatment might enhance specialized, β2-integrin-independent, transmigration of PMNs.

β2-integrin independent transmigration into alveoli during *Streptococcus pneumoniae* infection may also been mediated by galectin-3, a β-galactoside-binding protein released by alveolar resident macrophages, or pulmonary parenchyma cells [23]. This molecule promotes neutrophil adhesion to endothelium [23] and also affects other aspects of PMN function including transmigration [24] and superoxide anion production [25] (see below). In this study we found that IFN-treatment of PLB-985 cells caused a 10 fold increase in the mRNA for CD66a (CEACAM1), a major galectin-3 receptor present in neutrophils [26]. Thus the PLB-985 model suggests that IFN-γ could enhance β2-integrin independent PMN transmigration by upregulating PMN surface receptors that bind galectin-3. An additional neutrophil cell surface molecule involved in adherence is L-selectin which may facilitate stronger tethering by an already rolling neutrophil [27] and/or contribute to signaling that allows adherence and emigration out of blood vessels [28]. As shown in Table 1 L-selectin/CD62L (SELL) was upregulated 3.31 fold by IFN-γ treatment in this study; thus our model system indicating another possible mechanism by which IFN-γ might promote adherence in PMNs.

With respect to downstream intercellular signaling no clear conclusions can be drawn regarding the effect of IFN-γ on selectin and integrin signaling because although some potentially important components of the pathways [29] are down regulated, (Spleen Tyrosine Kinase (SYK) and the Src-kinase family member Frg (FRG)) others are up (the Src family kinase Lyn (LYN)). Since similar pathways act downstream of Fc-receptors and C-type lectins (see below) the same ambiguity applies to these neutrophil receptors as well.

#### Proteins involved in chemotaxis

After leaving the bloodstream, neutrophils move to sites of infection by chemotaxis along increasing gradients of chemoattractants molecules. Formylated bacterial peptides such as formyl-met-leu-phe (fMLF) are potent neutrophil -chemoattractants. These molecules bind to cell surface G-protein coupled receptors on PMNs and trigger various neutrophil responses including directed chemotaxis [30]. As shown in Table 1, we found that PLB-985 cell matured in the presence of IFN-γ had elevated mRNAs for two fMLF receptors (FPR1 and FPR2). Our model system thus reveals a potential mechanism by which IFN-γ treatment might alter PMN chemotaxis.

The soluble complement factor C3a, generated by the complement system at sites of infection, also triggers neutrophil chemotaxis. Although not as strong a chemoattractant as complement factor C5a, it is never the less a potent activator of PMN movement [31;32]. In our PLB-985 cell based model we found that IFN-γ treatment upregulated the mRNA for complement component 3a receptor 1 (C3AR1), a G-protein coupled C3a receptor (Table 1). This result from our model system thus reveals a potential mechanism by which IFN-γ might modulate C3a directed chemotaxis in neutrophils.

As mentioned in “Proteins Involved in Neutrophil Transmigration,” the mRNA for the galectin-3 receptor CD66a (CEACAM1, see Table 1) was upregulated in PLB-985 cells following IFN-γ treatment. Galectin-3 has been shown to be a chemoattractant for monocytes and macrophages [33]. Although no direct evidence exists for its effects on movement of neutrophils it is plausible that IFN-γ mediated upregulation of the galectin-3 receptor on PMNs could allow galectin-3 to act more potently as a PMN chemoattractant.

As discussed in “Proteins Involved in Neutrophil Transmigration,” the mRNA for the galectin-1 receptor (LGALS3BP) was also upregulated in PLB-985 cell following IFN-γ treatment. Because galectin-1 has been shown to act as a neutrophil chemoattractant [34] this from our model system suggests that IFN-γ induced upregulation of this receptor might enhance PMN chemotaxis by enhancing the response to galectin-1.

#### Proteins involved in phagocytosis

After encountering microorganisms, PMNs engulf them into phagolysosomal vacuoles. Initiation of phagocytosis is dependent on the presence of opsonins on the surface of microorganisms which are recognized by corresponding neutrophil receptors. Proteins of the complement system (the insoluble, membrane deposited, C3b and C3bi) and immunoglobulins are the main opsonins in serum. Immunoglobulins are recognized by Fc receptors (reviewed in [35–37]) and complement proteins are recognized by complement receptors.

In our PLB-985 cell based model (Table 1) we found that several Fc receptors that bind to IgGs were upregulated suggesting that IFN-γ treatment might enhance phagocytosis of immunoglobulin opsonized microorganisms by upregulating immunoglobulin receptors. The two high-affinity Fcγ receptors (FCGRIA and FCGRIB) were upregulated as was FcRγ (FCERIG) which associates with the high-affinity Fcγ receptors and is essential for their presence at the cell surface in a functional form. The phagocytosis-inducing FcγRIIA (FCGRIIA) was also upregulated. In contrast to the above receptors which promote phagocytosis, we also found upregulation of FcγRIIB (FCGR2B) which inhibits phagocytosis-promoting Fcγ receptors, therefore, although upregulation of phagocytosis-promoting IgG receptors predominated, estimating the overall effect of IFN-γ on phagocytosis must consider the effect of FcγRIIB. It should also be noted that Fc receptors can also activate processes such as the respiratory burst and degranulation, the observed upregulation of Fc receptors seen in our PLB-985 based model thus suggests a potential mechanism by which IFN-γ might enhance neutrophil microbial activity through phagocytosis-independent mechanisms. It is noteworthy that the IFN-γ induced upregulation of FCGRIA and FCGRIIA mRNAs and FCGRIA protein (Fig 2) which we observed is consistent with the protective effects of INF-γ on stored neutrophils [19]. This earlier work described IFN-γ mediated protection of the oxidative burst, bactericidal activity and adherence in stored neutrophils. This protection correlated with decreased reductions in FCGRIA and FCGRIIA mRNA and cell surface expression over time. Thus for FCGRIA and FCGRIIA there is consistency in the effect of IFN-γ between neutrophils and our model cells in two distinct experimental protocols.

The complement receptors CR3 and CR4 recognize the complement factor C3bi [38;39]. In the context of PMN function they also act as cell surface proteins involved in endothelial adhesion (see the section “Proteins Involved in Endothelial Adhesion”). As can be seen in Table 1, both of their corresponding α subunits (ITGAM and ITGAX respectively) were upregulated by IFN-γ treatment in PLB-985 cells. Taken together, the upregulation of CR3 and CR4 mRNAs in our cell culture based model suggest a potential mechanism by which IFN-γ might enhance ingestion of C3bi opsonized microorganisms by upregulating corresponding receptors on PMNs.

#### Proteins involved in killing of pathogens: Components of the NADPH oxidase, its upstream signaling molecules, myeloperoxidase and lysozyme

The Nox2/NADPH oxidase is a key microbicidal enzyme of neutrophils which generates a burst of reactive oxygen species (ROS) in response to microorganisms. It consists of a membrane associated heterodimer (cytochrome b558) of the proteins p22phox and gp91phox and the cytoplasmic proteins p40phox, p47phox and p67phox which bind to cytochrome b558 upon Nox2 activation. The resulting holoenzyme transfers electrons from cytoplasmic NADPH to extracellular O_2_ forming superoxide anion. Superoxide anion, and a cascade of ROS formed from it are, in large part responsible for the microbicidal activity of neutrophils [40].

Previously (12) we have described that application of IFN-γ to PLB985 cells during DMSO mediated maturation enhances fMLF and PMA stimulated superoxide anion production by the Nox2 enzyme. We also showed that this is accompanied by upregulation of mRNA (data reproduced in Table 1) for gp91phox (CYBB) and p47phox (NCF1) and corresponding increases in the levels of these proteins. The IFN-γ treatment was also seen to increase the level of p22phox but not its mRNA; because p22phox and gp91phox mutually co-stabilize each other, the observed increase in p22phox protein is most likely due to increased p22phox stabilization resulting from the increase in gp91phox. We also showed that IFN-γ treatment caused a large increase in membrane bound p22phox and gp91phox, i.e. an increase at an appropriate location for contributing to a functional Nox2 enzyme.

Activators of Nox2 include formylated peptides such as fMLF. As discussed in “Proteins Involved in Chemotaxis” these molecules are also chemoattractants for neutrophils. As shown in Table 1, we observed that IFN-γ treatment increased the levels of mRNAs for two different fMLF receptors in the PLB-985 model, those encoded by FPR1 and FPR2. This suggests a potential mechanism by which IFN-γ might enhance superoxide anion production triggered by formylated peptides neutrophils, i.e.due to up regulation of corresponding cell surface receptors.

As mentioned in the “Proteins Involved in Neutrophil Transmigration” section, mRNAs for the galectin-1 receptor (LGALS3BP, see Table 1) and the galectin-3 receptor CD66a (CEACAM1, see Table 1) were upregulated in PLB-985 cell following IFN-γ treatment. Both of these galectins have been shown to activate superoxide anion production by neutrophils [25;41] raising the possibility that IFN-γ treatment could enhance the binding of galectin-1 and -3 to their corresponding receptors on PMNs and thus promote Nox2 activity.

Lysozyme is a glycoside hydrolase that can damage bacterial cell walls. It is released from neutrophils and is transferred in to phagolysosomes in response to pathogens. It was upregulated modestly by IFN-γ treatment in our PLB-985 model (Table 1) which suggests another possible mechanism by which IFN-γ treatment might enhance the microbicidal activity of PMNs.

Superoxide anion is the initial ROS formed due to the activity of Nox2. It is converted to H_2_O_2_ either spontaneously or in a reaction catalyzed by superoxide dismutase. The H_2_O_2_ is then converted to hypohalous acid in a reaction catalyzed by myeloperoxidase (MPO). Hypochlorous acid is a potent microbicidal agent that is thought to play a role in PMN mediated microbe killing [42]. Counterintuitively, given the generally immune enhancing properties of IFN-γ, we found that IFN-γ treatment dramatically reduced expression of MPO mRNA (22 fold, Table 1). However, as reviewed in reference [42], even though MPO is involved in the microbicidal activity of normal neutrophils, MPO-independent antimicrobial systems are effective in MPO-deficient leukocytes, particularly when the microbial challenge is low. Indeed, because myeloperoxidase (MPO) deficiency is generally considered to be innocuous it has been removed as a primary immune deficiency disease as classified by the Primary Immunodeficiency Diseases Classification Committee of the International Union of Immunological Societies [43]. Another reason why IFN-γ induced MPO downregulation may not to significantly inhibit the microbicidal activity of PMNs is shown in Fig 2. Although myeloperoxidase -precursor protein was reduced dramatically, the level of heavy chain protein, which is produced from the precursor and is part of the active enzyme, was down less. This suggests that the active enzyme may be a more stable protein than the precursor and less susceptible to IFN-γ induced depletion in maturing neutrophils.

### Proteins involved in apoptosis and other mechanisms that regulate PMN concentration

Neutrophil blood count affects their ability to fight infection and is regulated by the balance of neutrophil release from the marrow and neutrophil apoptosis [44] as well as by other mechanisms such as neutrophil return to the marrow. Apoptosis is also involved in the efficient clearance of neutrophils and microorganisms that have ingested pathogens at a site of infection and provides for an orderly dissolution of the neutrophils without a release of their potentially damaging content [44]. The phagocytosis of certain bacteria by neutrophils accelerates apoptosis where as other bacteria inhibit normal phagocytosis-induced neutrophil apoptosis to evade being killed. This has led to the suggestion that bacteria-induced neutrophil apoptosis plays a role in the resolution of infection [45].

IFN-γ has been shown to increase the half-life of PMN in culture by protecting them from apoptotic cell death [46] suggesting that this cytokine is anti-apoptotic with respect to these cells.

In this study we find that the mRNAs of a number of proteins involved in apoptosis are altered by the presence of IFN-γ during PLB-985 maturation, however both potentially pro and anti-apoptotic changes are seen (Table 2). Given the experimental evidence for an anti-apoptotic effect of IFN-γ on PMN in culture the overall balance of the changes is apparently anti-apoptotic. The potential upregulation of pro-apoptotic proteins however might set the stage for rapid apoptosis and enhanced killing should microorganisms be encountered.

The mRNAs of pro-apoptotic proteins that are up regulated by IFN-γ include caspase 10 (CASP10), an initiator caspase that cleaves and activates the pro-forms of effector caspases (Table 2). Two effector caspases, caspase 7 (CASP7) and caspase 3 (CASP3), which cleave protein substrates during apoptosis, [46] were also seen to be upregulated (Table 2). Furthermore two subunits of a calpain protease, a large catalytic subunit (CAPN3) and a small subunit, calpain small subunit 2 (CAPNS2) were upregulated. Although these proteins have not specifically been implicated in caspase activation, other calpains have been shown to activate caspases [47] due to proteolysis of the anti-apoptotic protein, X-linked inhibitor of apoptosis (XIAP) and their upregulation might thus be pro-apoptotic. On the other hand calpastatin (CAST) was also upregulated and since it is a calpain inhibitor it is potentially anti-apoptotic.

The pro-apoptotic cell surface molecule, death receptor of the Fas ligand/CD95 (FAS) [48] was upregulated 5 fold by INF-γ treatment in our PLB-985 based model (Table 2). This molecule is potentially significant for neutrophil function because macrophages express high levels of Fas ligand on their cell surfaces; their arrival at a site of infection could thus lead to apoptotic clearance of bacteria-containing neutrophils via binding of neutrophil CD95 and macrophage Fas ligand. If Fas ligand is upregulated in PMNs as in PLB-985 cells then IFN-γ might enhance this process in vivo in people. Supporting this model, it has been shown that macrophages induce CD95 mediated neutrophil death [49]. Liver resident macrophages (Kupffer cells) are also important in PMN clearance and appear to also recognize PMN CD95 by means of surface expressed Fas ligand [50].

The cytokine tumor necrosis factor alpha (TNF-α) can regulate apoptosis in neutrophils [44;51]. Of the two TNF-α receptors (tumor necrosis factor receptors 1 and 2), we found that tumor necrosis receptor factor receptor 1 (TNFRSF1A) was upregulated by INF-γ treatment in PLB-985 cells. After engagement of TNF-α this receptor assembles an intracellular protein complex that activates anti-apoptotic signaling, however, in a second step, a component of this complex dissociates and participates in formation of a new caspase activating pro-apoptotoic complex, thus it is hard to determine whether TNFRSF1A upregulation is pro or anti-apoptotic.

The cytokine TNF-related apoptosis-inducing ligand/TRAIL (TNFSF10) also promotes apoptosis in neutrophils [51]. It was found to be upregulated dramatically (11 fold) by IFN-γ treatment in PLB-985 cells. This finding thus suggests a possible mechanism by which IFN-γ could alter neutrophil apoptosis via a change in autocrine, neutrophil-neutrophil, signaling by TRAIL.

As shown in Table 2, the B cell lymphoma-2 (Bcl-2) family of mitochondrial-membrane-associated-apoptotic-regulators [52] were regulated in both pro and anti-apoptotic ways; the pro-apoptotic Bax (BAX), Bak (BAK1), Bim (BCL2L11) and Bmf (BMF) proteins were upregulated but the anti-apoptotic members of the family Bcl-xL (BCL2L1), Mcl-1 (MCL1) and Bcl related protein A1 (BCL2A1) were also upregulated (in the case of Bcl-xL and Mcl-1 the picture is complicated by the fact that some their transcripts can be pro apoptotic). APOL6 encodes apolipoprotein L6 which was recently recognized as a proapoptotic Bcl-2 family member [53]. It was upregulated 7 fold by IFN-γ in PLB-985 cells this study.

Finally with respect to apoptosis related genes, although the mRNA for the apoptosis inhibitor XIAP was upregulated 1.55 fold, the mRNA for its pro-apoptotic inhibitor, inhibitor of XIAP (XAF1) was up dramatically (24 fold). XIAP binds to caspases 3, 7 and 9 and inhibits their proteolytic activity [54] whereas the XAF1 protein antagonizes this activity [55]. Our findings in PLB-985 cells could thus indicate a possible way in which IFN-γ might enhance apoptosis-assisted bacterial killing in neutrophils.

Another protein that is involved in PMN homeostasis is CXC-chemokine receptor 4 (CXCR4). PMNs can be removed from circulation by liver-resident Kupffer cells and also by return to the bone marrow. Increased expression of CXC-chemokine receptor 4 in older neutrophils is thought to enhance these processes [56]. CXC-chemokine receptor 4 is also thought to inhibit the release of newly matured PMN from bone marrow [57;58]. In this study, the mRNA encoding CXC-chemokine receptor 4 was down regulated by 2 fold (Table 2) by IFN-γtreatment in PLB-985 cells suggesting a potential mechanism by which IFN-γ might inhibit PMN clearance and promote PMN release from the bone marrow.

### Innate immune receptors

Neutrophils express a number of innate immune or pattern recognition receptors which bind to PAMPS, molecular signatures found in certain microorganisms and which are distinguishable from host molecules. Binding of these receptors to their corresponding pathogen-derived ligands triggers anti-microbicidal and pro-inflammatory changes in neutrophils that include production of pro-inflammatory cytokines, stimulation of phagocytosis and enhancement of the oxidative burst. We found that a number of these receptors were changed in our model system of PLB985 cells matured in the presence of IFN-γ relative to cells matured in its absence. This suggests potential mechanisms by which IFN-γ treatment might enhance neutrophil function by upregulating innate immune receptors and thus enhancing the anti-microbicidal changes in neutrophils that result from interaction of the receptors with their ligands.

#### Toll like receptors (TLRS)

TLRs are a family of transmembrane innate immune receptors found on many immune cells [59]. In neutrophils, all human TLRs, except TLR3, have been detected although the expression of TLR7 is controversial [29]. For PMNs, binding of TLRs to their corresponding ligands has been shown to trigger, or prime, cytokine release, superoxide generation, L-selectin shedding and phagocytosis of opsonized latex beads while inhibiting chemotaxis to interleukin-8 [60]. Delayed apoptosis induced by CpG motifs in bacterial DNA acting through TLR9 has also been observed [61]. Two subgroups of TLRs exist, one subgroup is located on cell surfaces and recognizes microbial membrane components whereas the other is on intracellular vesicles and recognizes microbial and viral nucleic acids.

As shown in Table 3, mRNAs encoding a number of TLRs, including examples from both TLR subgroups, were significantly altered in PLB-985 cells matured in the presence of IFN-γ relative to cells matured in its absence. TLR4, a founding member of the TLR family, forms a cell surface complex with the protein MD2 (LY96) which binds to bacterial lypopolysaccharide (LPS), a component of the outer membrane of Gram-negative bacteria [62]; strikingly both TLR4 and LY96 mRNA appeared to be elevated in PLB-985 cells treated with IFN-γ (2 and 6 fold respectively). Our findings in PLB-985 cells thus indicate a potential mechanism by which INF-γ treatment might enhance the PMN response to LPS.

The mRNA for TLR8, which recognizes nucleic acid ligands including G-rich oligonucleotides [63], was upregulated 6 fold in our PLB-985 based model. Although TLR8 is thought to recognize single stranded viral RNA [62], which is not relevant for neutrophils, it is phylogenetically most similar to TLR7 [62] which can sense RNA species from bacteria such as group B *Streptococcus* [64]; similar recognition of bacterial RNA by TLR8 might thus be important for the anti-bactericidal activity of PMNs.

TLR1 and TLR2 form a heterodimer which recognizes peptidoglycan and triacyl lipoprotein from Gram-negative bacteria and mycoplasma [62]; although TLR1 was up regulated, TLR2 was down regulated and thus the significance of these changes is unclear.

TLR3 was somewhat elevated by INF-γ treatment in PLB-985 cells but, like TLR8, it is thought to recognize viral RNA species and thus its relevance to PMN function is unclear [62].

TLR9 has been shown to inhibit neutrophil apoptosis in response to CpG motifs in bacterial DNA [61]. The IFN-γ induced down regulation of TLR9 which we observed in PLB-985 cells may thus reduce neutrophil lifespans in the presence of bacterial DNA. However, as described earlier, bacteria-induced neutrophil apoptosis may assist in clearance of infections [44;45] and if TLR9 downregulation in neutrophils reflects what we observed in our model system it could support the microbicidal activity of neutrophils by promoting their apoptosis in the presence of bacterial DNA.

Binding of TLRs to their ligands activates signaling pathways that trigger the corresponding neutrophil responses [29]. The MYD88 gene product is an adapter protein involved early in these pathways and we found it to be upregulated by IFN-γ in maturing PLB-985 cells. Furthermore, additional genes involved in signaling downstream of TLR receptors (RIPK1, RIPK2, TANK, and TRAF6) were also found to be upregulated by IFN-γ and the IRAK3 [65] gene, which encodes a negative regulator of TLR signaling was down regulated. Thus, upregulation of signaling downstream from TLRs may also allow IFN-γ to enhance neutrophil function.

#### Nod-like receptors and inflammasomes

Nod-like receptors are cytoplasmic innate immune receptors. Binding of Nod-like receptors to their corresponding ligands promotes pro-inflammatory cytokine production by means of transcriptional changes and activation of cytokine-processing caspases [29].

The Nod-like receptors NOD 1 and NOD2 bind to proteoglycan degradation products derived from bacteria [66]. In a previously study, NOD1 was undetected on PMNs [67], however we find that IFN-γ application during maturation leads to upregulation of its mRNA in PLB-985 cells (Table 3) suggesting that IFN-γ might allow pattern recognition by this molecule to be switched on in neutrophils. NOD2, which has been described previously in PMNs (67), was not upregulated by IFN-γ treatment.

The NOD-like NLRP3 receptor (NLRP3) is part of the NLRP3 inflammasome. Its binding to bacterial molecules activates the inflammasome and promotes conversion of pro-IL1β and pro- IL18 to their active forms due to proteolytic cleavage mediated by the inflammasome subunit caspase 1 [68;69]. We found that NLRP3 mRNA was up regulated 1.78 fold by the presence of INF-γ during PLB-985 maturation (Table 3). This finding is more striking given that, as shown in Table 3, two other NLRP3 inflammasome components, caspase 1 (CASP1) and the protein ASC (PYCARD) were also elevated by IFN-γ treatment. This suggests that, if our PLB-985 based observations are reflected in PMNs, then IFN-γ treatment could enhance NLRP3 inflammasome activity by increasing expression of its components and could thus up regulate production of IL1β and IL18 in response to inflammasome activation. All the essential components of the NLRP3 inflammasome have been previously described in neutrophils [70].

ASC has also been found to stimulate caspase 1 activity and IL1β production, in response to cytosolic dsDNA, in an NLRP3 independent manner [71]. In this case, the protein AIM2 (AIM2) acts as an innate immune receptor that binds dsDNA and then associates with ASC to activate caspase 1. This process is activated by *Listeria monocytogenes* that escapes from the phagolysosome of macrophages [72] and may thus also be important in PMNs. We found that IFN-γ treatment increased AIM2 mRNA expression dramatically (25 fold, Table 3) in PLB-985 cells. AIM2 upregulation in conjunction with ASC upregulation could therefore be an additional mechanism by which IFN-γ-might enhance cytokine production in PMNs.

#### C-type lectins

C-type lectins bind to carbohydrates in a Ca^2+^ dependent manner and, in vertebrates, members of the family are innate immune receptors [73]. Many C-type lectins are expressed in PMNs [29]. We find that IFN-γ applied during PLB-985 maturation causes changes in the levels of several C-type lectins relative to cells matured in the absence of IFN-γ (Table 3) which may be reflected in PMNs exposed to IFN-γ.

Dectin 1 (CLEC7A) is known to be expressed in PMNs [74] and was upregulated in maturing PLB-985 cells upon by IFN-γ treatment (Table 3). It recognizes 1,3-linked β-glucans found on some fungal, plant and bacterial cell walls [75]. This is potentially significant for neutrophils in which β-glucan binding by dectin 1 can activate the respiratory burst, ligand uptake by endocytosis and phagocytosis, the production of arachidonic acid metabolites and cytokine and chemokine production [76].

MICL (CLEC12A) is predominantly expressed by myeloid cells including PMNs [77] and its expression is down regulated during microbe-induced inflammation [78]. Its cytoplasmic tail has been shown to inhibit TNF-α production and phagocytosis of [77]. The observed downregulation of CLEC12A by IFN-γ in PLB-985 cells, if mirrored in PMNs, could potentially be significant for PMN function as release of CLEC12A mediated inhibition might allow increased TNF-α production and phagocytosis of particulate stimuli by PMN.

The C-type lectins mincle receptor (CLEC4E) and MCL (CLEC4D) bind to cord factor, a glycolipid found on *M*. *tuberculosis* cells. Expression of mincle receptor on PMNs is thought to be crucial for PMN infiltration of *M*. *tuberculosis-induced* lung granulomas. Additionally, co-activation of Mincle and TLR2 pathways synergistically induces CD11b surface expression, reactive oxygen species production and TNF-α production by neutrophils [79]. Thus the down regulation of TLR2, CLEC4E and CLEC4D (Table 3) by IFN-γ in PLB-985 cells, if also seem in PMNs, may potentially reduce the microbicidal and proinflammatory activity of neutrophils in response to *M*. *tuberculosis*.

Little information about the role of CLEC2B and CLEC5A, which were respectively up and downregulated by IFN-γ (Table 3) is available.

In summary, the changes in C-type lectin expression mediate by IFN-γ in PLB-985 cells could potentially both increase and decrease microbicidal and proinflammatory activity if also observed in neutrophils. Whether IFN-γ supports or inhibits the immune activity of neutrophils via changes C-type lectin expression may thus depend on which pathogen is being targeted and which PAMPs are associated with it. For example, IFN-γ might enhance the neutrophil response to pathogens associated with 1,3-linked β-glucans due to upregulation of CLEC7A but inhibit the response to *M*. *tuberculosis* due to inhibition of CLEC4D and CLEC4E expression.

### Guanylate binding proteins

Expression of certain members of the Gbp family is known to be enhanced by IFN-γ [80]. As shown in Table 4, in the current study, Gbp mRNAs were strongly upregulated in PLB-985 cells matured in the presence of IFN-γ relative to those matured in its absence. Antiviral or antibacterial activities have been ascribed to Gbps 1, 2, 3, 5 and 6, which were upregulated by INF-γ in our study, and to Gbps 7 and 10 which were not upregulated [81–84].

We found that the mRNAs for Gbp 1(GBP1) and Gbp2 (GBP2) were up regulated 188 and 213 fold respectively in PLB-985 cells matured in the presence of IFN-γ relative to cells matured in its absence. These proteins have been shown to inhibit the growth of *Chlamydia trachomatis* in HeLa cells [83]. The growth inhibition was dependent on their GTPase domains and both proteins were shown to localize to inclusion membranes surrounding internalized chlamydiae [83].

In a different study Gbp1 was found to inhibit growth of *Listeria monocytogenes* and *Mycobacterium bovis* in RAW264.7 macrophages [82]. Gbp6 (GBP6), which was up 2.46 fold with IFN-γ treatment of PLB-985 cells in this study, was also found to inhibit these pathogens. Again, translocation of Gbps to bacteria containing vacuoles was observed. For Gbp1 the translocation was shown to involve co-transport of the protein p62/Sqstm1, which itself carries ubiqitinated proteins; this was found to generate ubiquitin derived microbicidal peptides in mycobacteria-containing vacuoles [82].

Since our model system suggests that Gbps 1,2 and 6 might be upregulated in PMNs following IFN-γ treatment their bacteriostatic activity in other cell types represents a potential mechanism by which IFN-γ could enhance PMN function.

Although Gbp7 was not up regulated in our study it was shown to co-transport components of the bactericidal NADPH oxidase (p22phox, gp91phox and p67phox) to bacteria-containing vesicles [82]. We propose that some of the Gbps upregulated in this study could also function to transport phox proteins to the phagolysosome in neutrophils and that this could potentially explain some of the immune supportive effect of IFN-γ with respect to neutrophils.

We found the mRNA encoding Gbp5 (GBP5) to be strongly upregulated (337 fold) by IFN-γ in our PLB-985 based model. In mouse and human macrophages Gbp5 has been shown to promote NLRP3 inflammasome assembly and activity in response to pathogenic bacteria and soluble inflammasome priming agents [81]; Gbp5 was necessary for proteolytic activation of the inflammasome component caspase 1 and for the caspase 1 mediated cleavage of pro-IL1β to give active IL1β. Furthermore, tetramers of Gbp5 were shown to promote inflammasome assembly. Our current observation that Gbp5 was upregulated by IFN-γ treatment is thus all the more striking given that we also found three other components of this inflammasome (see Table 4) to be up regulated by IFN-γ treatment. Based on our findings from the PLB-985 model this evidence in the literature suggest that IFN-γ applied to neutrophils might enhance NLRP3 inflammasome assembly and IL1β production via Gbp5 upregulation in a manner similar to that described for macrophages.

To our knowledge there is no evidence related to the role of Gbps in neutrophils. However they might have functions in these cells that are similar to those outlined above for other cell types. Therefore, extrapolating from our model system, IFN-γmight enhance neutrophil mediated immunity by increasing Gbp expression and upregulating the immune supportive roles of these GTPases in these cells.

### Proteins involved, or potentially involved, in antigen presentation by MHCII

Members of the MHCII receptor family are transmembrane proteins that occur on antigen presenting cells (APCs) which are generally considered to be dendritic cells, macrophages and certain B-cells. APCs process antigens into peptide fragments that are presented on the extracellular portions of the MHCII receptors. T-cells with T-cell-receptors that recognize the presented peptide interact with the corresponding APC which leads to T-cell proliferation and differentiation [85].

PMNs have classically been considered as exclusively involved in innate immunity and to have no role in antigen presentation or T-cell activation. However a number of studies have shown that PMNs can express MHCII molecules on their surfaces when treated with pro-inflammatory stimuli including IFN-γ [86–88]. These expressed MHCII molecules appear to be functional since PMNs have been shown to mediate T-cell activation in response to staphylococcal enterotoxin, [87] a superantigen that can be presented on MHCII receptors without intracellular processing. With respect to whether PMNs can mediate full antigen processing by the standard pathway there are conflicting results, in the initial study showing presentation of staphylococcal enterotoxin PMNS were found to not process the tetanus toxoid antigen [87] but in subsequent work they were found to activate T-cells after MHCII induction and tetanus toxoid treatment [86]. Genetic polymorphism in human populations could explain these discrepancies since only 51% of human subjects were found to have PMNs that could be induced to express MHCII [89].

In this study, IFN-γ treatment of PLB-985 cells was found to upregulate the mRNAs encoding proteins involved in MHCII antigen presentation which are discussed in more detail below. Our model system is thus consistent with previous studies indicating that IFN-γ induces expression of MHCII molecules and also extends the number of MHCII components known to be modulated by it. Extrapolating our findings to PMNs thus supports this idea that one way in which IFN-γ might boost neutrophil mediated immunity is by enhancing a little appreciated interaction between PMNs and adaptive immunity. The relevant proteins are discussed below.

We found a 6 fold upregulation (Table 5) of the mRNA encoding class II transactivator (CIITA) which is the "master control factor" for the expression of MHCII genes [90]. Class II transactivator mainly stimulates transcription of genes involved in the presentation of antigenic peptides by MHCII molecules and its expression is thought to be the most important factor governing MHCII expression [90]. Class II transactivator, when bound to GTP [91], enters the nucleus and activates transcription of its target genes via activation of the transcription factor DNA-binding protein RFX5 (RFX5) which itself was upregulated by IFN-γ treatment, in PLB-985 cells, this study (Table 5).

The three different MHCII receptors, HLA-DQ, HLA-DR and HLA-DP are heterodimers of different α and β proteins. In our model system we found that IFN-γ induced dramatic (75 fold) upregulation of the mRNA encoding the single α subunit of HLA-DR (HLA-DRA) but not any of its β subunits (Table 5). We also found upregulation of the mRNAs encoding the single α subunit of HLA-DP (HLA-DPA1) as well as its single β subunit (HLA-DPB1).

Invariant chain/Ii/CD74 (CD74) is a crucial component of the MHCII system. Immediately after synthesis in the endoplasmic reticulum, new MHCII receptors bind to CD74 and a portion of CD74 occupies their processed-antigen-binding-sites to prevent premature peptide entry. In this study we found the mRNA encoding CD74 to undergo a large upregulation (21 fold) upon IFN-γ treatment (Table 5).

HLA-DM is a transmembrane heterodimer that is highly homologous to MHCII receptors. It removes CD74 from MHCII receptors in antigen processing compartments of APCs [85]. We found that mRNA for both its α and β subunits (HLA-DMA and HLA-DMB respectively) were upregulated by IFN-γ treatment (Table 5) in our PLB-985 model.

APC interaction with T cells, and T-cell activation, is mediated by the binding of APC MHCII receptors to T-cell receptors that recognize the corresponding presented antigen but is further enhanced by co-receptor interactions between T-cells and APCs; an example is the interaction of APC CD86 and T-cell CD28 [92]. We found that IFN-γ treatment increased expression of the mRNA for CD86 by 1.5 fold (Table 5) in PLB-985 cells. If PMNs can act as APCs then co-receptor interactions might be an important part of that process and our data suggests that IFN-γ might enhance CD86-CD28 binding by upregulating CD86. Similar co-receptors interactions also come into play when B cells interact with T cells, for example B cell CD40 binding to T cell CD40 ligand [93]. In this study we found that CD40 in PLB-985 cells was upregulated 12 fold by IFN-γ suggesting another potential PMN-T cell co-receptor interaction. Another APC protein which may act in co-receptor interactions with T-cells is encoded by PDCD1LG2, the corresponding mRNA was upregulated dramatically (12 fold) by IFN-γ in our PLB-985 cell model (Table 5) but any putative role in PMN mediated T-cell activation is obscured by the fact that controversy exists regarding whether it is T-cell stimulatory or inhibitory [94]. In contrast to the T-cell-activating co-receptor interactions described above we found that IFN-γ increased expression of PDCD1LG1/PDL1 (CD274) mRNA by 36 fold and interaction of this molecule with its T-cell receptor inhibits T-cell activation and cytokine production [95]. Thus in this study both potential T-cell enhancing and inhibiting co-receptors are upregulated making it hard to predict the effect of any possible effect of net T-cell activation based simply on gene expression. However, the finding that neutrophils activate T-cells after MHCII induction and tetanus toxoid treatment [86] suggests that, on balance, the net effect of IFN-γ is toward T-cell activation.

Processing of antigens in APCs occurs in lysosomes and is carried out by lysosomal proteases of the cathepsin family. A number of cathepsins (discussed below) were upregulated by IFN-γ treatment in our PLB-985 model. This thus suggests another possible mechanism by which IFN-γ might enhance the MHCII system in neutrophils. Cathepsin B, D, E, L, S, V and W have been implicated in antigen processing for MHCII (reviewed in [96]). Of these, we found that mRNAs for cathepsins B (CTSB), L (CTSL1) and S (CTSS) were upregulated by IFN-γ treatment in the PLB-985 cell model system (Table 5). Furthermore, mRNAs for cathepsins O (CTSO), Z (CTSZ) and A (CTSA) were upregulated; since little is known about the roles of these proteases it is plausible that they might also be involved in processing antigens for MHCII presentation.

Collectively the findings outlined in this section support the notion that PMNs could act as APCs because various molecular players of antigen presentation by MHCII appear to be upregulated by IFN-γ, at least in our current model. This is consistent with previous results from work done using PMNS [86–88]. Enhancement of this process in PMNs could contribute to the ability of IFN-γ to support the immune system in people to who it is administered.

### Proteins involved in antigen presentation by MHCI

Some evidence suggests that neutrophils also play a role in adaptive immunity via the MHCI system [97;98]. In contrast to MHCII, MHCI molecules are widespread and are found on most cell types. They are transmembrane proteins which present non-self-protein fragments from within a cell at the cell surface; this allow the presenting cells to interact with T cells expressing T-cell receptors corresponding to the presented antigen and leads to T-cell activation. Circulating neutrophils express MHCI molecules [99–101] and it has been shown that peptides added to PMN are presented on cell surface MHCI molecules and can thus stimulate CD8 positive memory T cells [98]. Peptides presented by MHCI are derived from cytoplasmic proteins which are either degraded by proteasomes or processed with in vacuolar compartments independent of proteasome activity; MHCI processing in neutrophils has been shown to be mediated by a vacuolar rather than cytoplasmic pathway [97].

MHCI molecules are composed of cell surface heterodimers consisting of one of several α chains bound non-covalently to the protein β2-microglobulin/b2m [102]. As shown in Table 5, we found that multiple MHCI α chains were upregulated by IFN-γ (HLA-A, HLA-B, HLA-C, HLA-E, HLA-F and HLA-G) as was b2m (B2M) in our PLB-985 model system. Extrapolating our findings to neutrophils, this suggests that their role in adaptive immunity might be enhanced when they are matured in the presence of IFN-γ because of increased expression of MHCI heterodimers.

In the endoplasmic reticulum non-self-peptides are loaded on to MHCI molecules by a protein complex composed of the following proteins: transporter associated with antigen presentation (TAP), Tap1, Tap2, tapasin, ERp57 and calreticulin [102]. This complex, the peptide loading complex (PLC), also functions to partially fold and stabilize MHCI proteins. As shown in Table 5, we found upregulation of mRNAs encoding Tap1 (TAP1), Tap2 (TAP2), Tapasin (TAPBP) and Erp57 (PDIA3) and a relatively much smaller down regulation of calreticulin (CALR) in our model sytem. Thus, on balance, the data suggests that IFN-γ might also enhance peptide presentation by MHCI due to increased expression of the PLC.

The role of the TAP protein within the PLC is transportation of proteasome derived cytoplasmic peptides into the endoplasmic reticulum. The previous observation that MHCI presentation in neutrophils does not involve cytoplasmic processing [97] was made using non-IFN-γ-treated cells and therefore our finding that TAP expression is upregulated by IFN-γ hints that this cytokine could enhance the MHCI system in PMNs by promoting presentation of proteasome derived peptides of cytoplasmic origin.

Our data indicating that IFN-γ enhances expression of proteins involved in the MHCI system in PLB-985 cells suggest that a similar effect might be seen in vivo in PMNs. This supports the idea that IFN-γ supports immune function due, in part, to enhancement of MHCI based antigen presentation by neutrophils. They are also consistent with the idea that neutrophils might have a role in adaptive immunity [97;98] due to antigen presentation by MHCI.

### Caveats related to the use of PLB-985 cells

PLB-985 cells are a myeloid cell line that are a subclone of the HL-60 cell line [103] but which have characteristics consistent with a more primitive precursor along the myeloid lineage [11;12;104]; PLB985 cells are more myeloblast-like while HL60 cells are more promyeloctic/myelocytic. While DMSO differentiated PLB-985 and HL-60 cells have been widely as a neutrophil-like model, their similarity to cells early in the myeloid lineage suggests that some of the findings from this study might relate more closely to effects that IFN-γ could have on monocytes and/or other phagocytes rather than on neutrophils. Studies of the effects of IFN-γ on different phagocytes from human subjects treated with IFN-γ are necessary to address these uncertainties.

### Comparison of the effects of IFN-γ on immune related gene expression in maturing and non-maturing cells

As shown in Fig 2 and described in Results, when IFN-γ induced large expression changes in maturing cells, the corresponding changes in non-maturing cells were much smaller.

Potential reasons why IFN-γ might cause larger and more extensive expression changes in maturing cells include a mechanism where by the developmental changes related to differentiation relieve transcriptional repression of certain genes and thus enhances the ability of IFN-γ to modulate their expression. Another possibility is that PLB-985 maturation leads to expression of proteins that are in signaling networks that transduce IFN-γ interactions with its cell surface receptors (interferon-γ receptors 1 and 2) to the transcription of certain genes.

The fact that many of the immune related genes of interest identified in this study are much more strongly affected by IFN-γ applied to maturing cells validates our use of differentiating PLB-985 cells as a model. It indicates that many of the gene changes we observed are the result of biological interactions between differentiation and IFN-γ and are not simply due to the effect of prolonged application of the cytokine to PLB-985 cells in culture. Many of the gene expression changes observed would have been much smaller if IFN-γ had simply been applied for 72 hours to non-maturing PLB-985 cultures. On the other hand, the data in Fig 2 also indicated that for many genes (particularly those in Fig 2C) expression changes seen in maturing cells were very close to those caused by IFN-γ alone. For these genes, application of the cytokine for 3 days appears to account for most of the changes in expression and ongoing cell differentiation does not appear to play a role.

### Comparison of the effects of IFN-γ on immune related gene expression in maturing and already mature cells

As shown in Table 6, for some genes, similar changes in expression were observed when IFN-γ was applied during the 72 hour DMSO-induced maturation period and when it was applied to already matured cells for 3 hours. In contrast, for other genes much more dramatic changes were dependent on IFN-γ application throughout the maturation period.

This latter group of genes further validates our use of differentiating PLB-985 cells as a model. It indicates that many of the gene changes we observed are the result of biological interactions between differentiation and IFN-γ and are not simply due to the effect of application of the cytokine to mature, neutrophil-like, PLB-985 cells in culture.

## Conclusion

The model system used in this study to explore the effect of IFN-γ on maturing neutrophils identified many gene expression changes that could account for the improved immune function of CGD patients taking the drug. These individuals lack proteins that are components of the NADPH oxidase enzyme complex which mediates production of bactericidal ROS by neutrophils. Although IFN-γ enhances expression of these proteins in people without CGD [5–8] it obviously cannot enhance expression of oxidase proteins in individuals with gene defects that completely abolish expression of these proteins; nevertheless use of IFN-γ helps reduce serious infection in such individuals [4]. Based on the gene expression changes observed we propose that neutrophil function in cells lacking the NADPH oxidase could be the result of enhancement of other aspects of PMN function. Cells that have enchaned transmigration, chemotaxis and ingestion could show increased killing via more rapid and extensive bacterial sequestration in the phagalysosome and thus enhanced non-oxidative killing in this compartment, i.e. that mediated by bactericidal components of granules such as lactoferrin and lysozyme (related to this point, lysozyme expression was enhanced by IFN-γ in this study, see Table 1). By modulating neutrophil clearance and homeostasis the drug might enhance the life span of neutrophils and promote more efficient apoptosis after bacterial ingestion which is thought to be part of efficient killing [45]. IFN-γ induced upregulation of innate immune receptors will not enhance ROS production in CGD neutrophils but might enhance production of pro-inflammatory cytokines [29;59;73] that could functionally support other cells of the innate immune system and could also improve neutrophil ingestion and corresponding non-oxidative killing by neutrophils. Upregulation of GBPs by IFN-γ could mediate non-oxidative killing via known bactericidal activities of this family of proteins (e.g. the generation of microbicidal peptides in the phagolysosome [82]) and as yet unknown mechanisms by which GBPs exert their generally bactericidal/bacteroiostatic functions. Finally, although a role for neutrophils in adaptive immunity is controversial some evidence supports it [86–88;97;98] and enhancement of an MHCI and II mediated role for PMNs in adaptive immunity might benefit CGD patients incapable of making certain NADPH oxidase proteins. The identification of the above gene expression changes in PLB-985 cells provides a valuable framework for examining gene expression changes in neutrophils, other granulocytes, monocytes and other immune cells in human subjects given IFN-γ.

## Supporting information

S1 Fig Full length images of the blots shown in Fig 1. The entire lanes of the blots which are cropped in Fig 1 are shown. The bands corresponding to the proteins of interest are indicated by the corresponding gene name. In the cases of multiple bands or broad bands the proteins of interest are indicated by horizontal lines, the details of such cases are in Fig 1. The sizes of the bands of interest correspond well with the calculated sizes (based on amino acid sequence) of the proteins or example blots from the relevant antibody manufacturers. The one exception is HLA-DRA which is much larger than expected. However, western blots for HLA-DRA from HL-60 cells (of which PLB-985 cells are a subclone [12]) have previously shown a larger than expected band of immune staining [105]. This was tentatively attributed to a reducing and boiling-resistant association of HLA-DRA and DRB proteins. Unlike the other blots in Fig 1, given the unexpected size of the band on the HLA-DRA blot, we are somewhat cautious about using the HLA-DRA blot as an independent example of protein expression matching mRNA expression in this study.
(TIF)

S1 Table Comparison of mRNA changes caused by IFN-γ application to already mature cells and IFN-γ application during cell maturation. mRNA changes caused by 3 hour applications of IFN-γ to already mature cells are in the column “Fold change for mature cells treated with IFN-γ versus untreated mature cells”. The corresponding ANOVA p-values are also shown. For comparison, the mRNA changes from Tables 1–5 that were caused by IFN-γ application during DMSO mediated differentiation are in the column “Fold change for DMSO plus IFN-γ treatment versus DMSO treatment”.
(DOCX)
