# Supplementary figures and images for: IFN-γ alters the expression of diverse immunity related genes in a cell culture model designed to represent maturing neutrophils
Source: PLoS One. 2017 Oct 5;12(10):e0185956. doi: 10.1371/journal.pone.0185956 (PMC5628906; doi:10.1371/journal.pone.0185956)

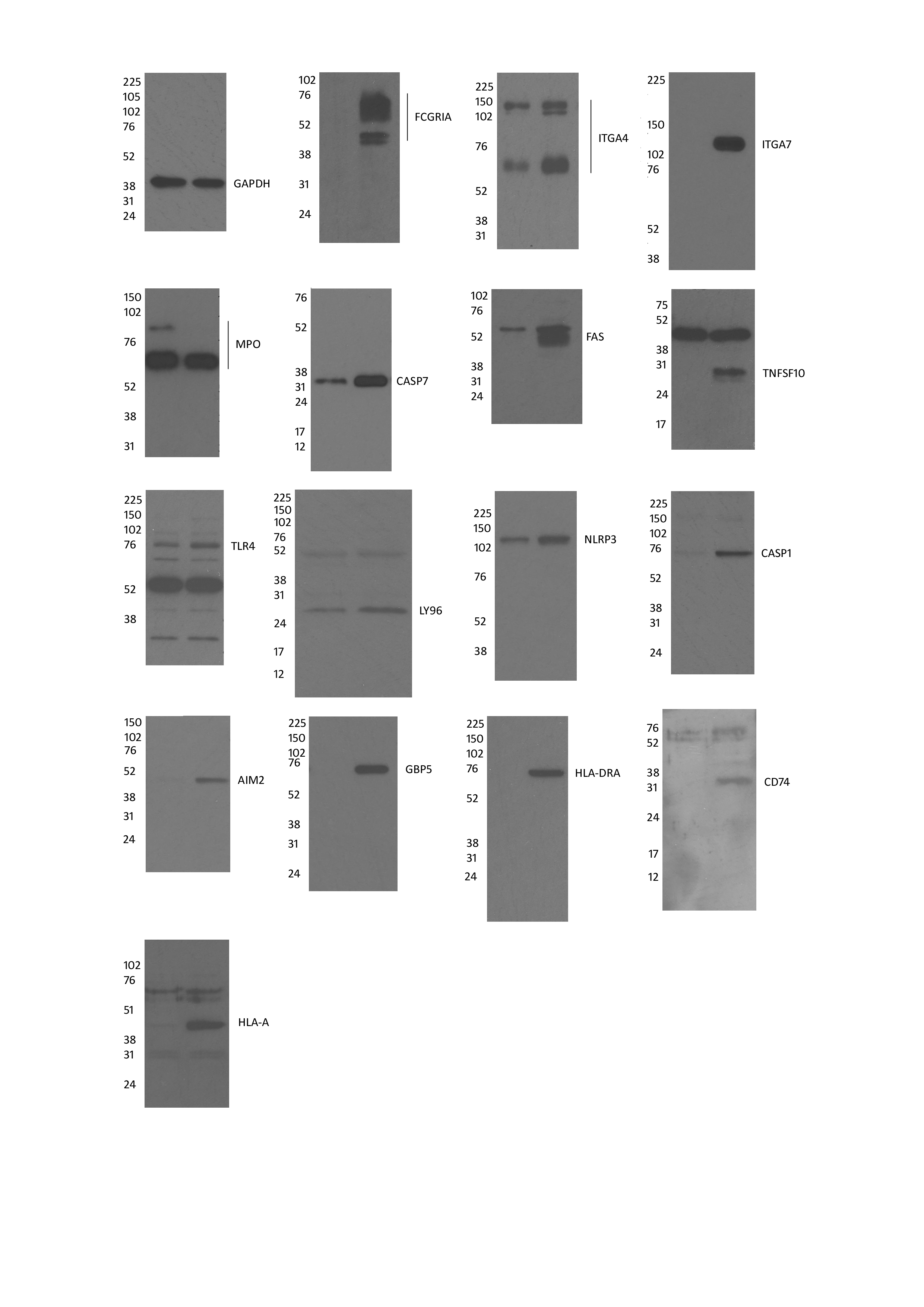

Supplement: S1 Fig — The entire lanes of the blots which are cropped in Fig 1 are shown. The bands corresponding to the proteins of interest are indicated by the corresponding gene name. In the cases of multiple bands or broad bands the proteins of interest are indicated by horizontal lines, the details of such cases are in Fig 1. The sizes of the bands of interest correspond well with the calculated sizes (based on amino acid sequence) of the proteins or example blots from the relevant antibody manufacturers. The one exception is HLA-DRA which is much larger than expected. However, western blots for HLA-DRA from HL-60 cells (of which PLB-985 cells are a subclone [12]) have previously shown a larger than expected band of immune staining [105]. This was tentatively attributed to a reducing and boiling-resistant association of HLA-DRA and DRB proteins. Unlike the other blots in Fig 1, given the unexpected size of the band on the HLA-DRA blot, we are somewhat cautious about using the HLA-DRA blot as an independent example of protein expression matching mRNA expression in this study. (TIF) [file pone.0185956.s001.tif]
