# Supplementary material for: IFN-γ alters the expression of diverse immunity related genes in a cell culture model designed to represent maturing neutrophils
Source: PLoS One. 2017 Oct 5;12(10):e0185956. doi: 10.1371/journal.pone.0185956 (PMC5628906; doi:10.1371/journal.pone.0185956)
Supplement: S1 Table — mRNA changes caused by 3 hour applications of IFN-γ to already mature cells are in the column “Fold change for mature cells treated with IFN-γ versus untreated mature cells”. The corresponding ANOVA p-values are also shown. For comparison, the mRNA changes from Tables 1–5 that were caused by IFN-γ application during DMSO mediated differentiation are in the column “Fold change for DMSO plus IFN-γ treatment versus DMSO treatment”. (DOCX) [file pone.0185956.s002.docx]

| Gene | Fold change for mature cells treated with IFN-γ versus untreated mature cells | ANOVA p-value | Fold change for DMSO plus IFN-γ treatment versus DMSO treatment |
| --- | --- | --- | --- |
| SELPLG | 1.26 | 0.001993 | 1.21 |
| ITGAM | 1.14 | 0.002729 | 1.32 |
| ITGAL | -1.42 | 0.000048 | 1.84 |
| ITGAX | -1.35 | 0.017433 | 1.33 |
| ITGA4 | 1.7 | 0.000166 | 2.13 |
| ITGB7 | 1.47 | 0.013479 | 6.03 |
| CEACAM1 | 2.31 | 0.000983 | 10.42 |
| SELL | -1.01 | 0.69664 | 3.31 |
| SYK | -1.29 | 0.000215 | -1.4 |
| FGR | -1.34 | 0.107977 | -1.72 |
| LYN | 1.08 | 0.090108 | 1.47 |
| FPR1 | 1.36 | 0.000697 | 1.66 |
| FPR2 | 1.14 | 0.09847 | 1.48 |
| C3AR1 | -1.13 | 0.354762 | 1.72 |
| FCGR1A | 22.57 | 8.44E-08 | 39.77 |
| FCGR1B | 5.94 | 0.000027 | 5.33 |
| FCGR2A | -1.11 | 0.611421 | 1.81 |
| FCGR2B | -1.02 | 0.847736 | 2.12 |
| FCER1G | -1.02 | 0.729799 | 1.48 |
| CYBB* | 1.11 | 0.135054 | 1.19 |
| NCF1* | 1.03 | 0.434032 | 1.51 |
| LYZ | -1.16 | 0.053087 | 1.3 |
| MPO | -1.05 | 0.912466 | -22.24 |
| CASP10 | 1.89 | 0.000039 | 3.05 |
| CASP7 | 2.8 | 0.000002 | 2.3 |
| CASP3 | 1.65 | 0.000187 | 1.73 |
| CAPN3 | 1.11 | 0.903411 | 2.04 |
| CAPNS2 | 1.38 | 0.027137 | 1.64 |
| CAST | -1.15 | 0.025676 | 1.39 |
| FAS | 6.97 | 0.000003 | 5.35 |
| TNFRSF1A | 1.55 | 0.001001 | 1.31 |
| TNFSF10 | 10.23 | 6.85E-09 | 10.59 |
| BAX | 1 | 0.639238 | 1.29 |
| BAK1 | 1.58 | 0.000289 | 2.05 |
| BCL2L11 | 1.9 | 0.000149 | 1.38 |
| BMF | -1.14 | 0.02221 | 1.24 |
| BCL2L1 | 1.6 | 0.006036 | 1.62 |
| MCL1 | 1.49 | 0.00136 | 1.69 |
| BCL2A1 | 1.07 | 0.0202419 | 1.56 |
| APOL6 | 7.65 | 3.28E-08 | 6.6 |
| XIAP | 1.4 | 0.01932 | 1.55 |
| XAF1 | 1.12 | 0.143187 | 23.45 |
| CXCR4 | -1.75 | 0.000002 | -1.98 |
| TLR4 | 1.71 | 0.000222 | 2.14 |
| LY96 | 2.04 | 0.00036 | 5.96 |
| TLR8 | 3.55 | 0.0000009 | 5.5 |
| TLR1 | 1.49 | 0.000125 | 2.1 |
| TLR2 | 1.02 | 0.888932 | -1.5 |
| TLR3 | 1.04 | 0.51832 | 1.44 |
| TLR9 | -1.2 | 0.016922 | -1.28 |
| MYD88 | 1.35 | 0.001352 | 1.48 |
| RIPK1 | 1.43 | 0.001211 | 1.28 |
| RIPK2 | 2.15 | 1.4E-07 | 2.6 |
| TANK | 1.3 | 0.00255 | 1.48 |
| TRAF6 | 1.55 | 0.000241 | 1.31 |
| IRAK3 | -1.41 | 0.000047 | -1.59 |
| NOD1 | 1.16 | 0.050955 | 1.38 |
| NLRP3 | -1.54 | 0.010037 | 1.78 |
| CASP1 | 3.2 | 0.000001 | 5 |
| PYCARD | -1.1 | 0.279478 | 1.37 |
| AIM2 | 1.59 | 0.001082 | 24.56 |
| CLEC7A | -1.23 | 0.269696 | 1.6 |
| CLEC12A | -1.16 | 0.058255 | -2.23 |
| CLEC4E | -1 | 0.856575 | -1.43 |
| CLEC4D | -1.55 | 0.011814 | -1.55 |
| CLEC2B | 8.15 | 0.000001 | 8.15 |
| CLEC5A | -2 | 0.018912 | -2.4 |
| GBP5 | 13.36 | 6.32E-07 | 337.28 |
| GBP4 | 10.38 | 0.000005 | 248.98 |
| GBP2 | 95.45 | 1.9E-09 | 213.09 |
| GBP1 | 23.09 | 3.68E-07 | 188.25 |
| GBP3 | 15.77 | 3.14E-07 | 22.21 |
| GBP6 | 1.1 | 0.282663 | 2.43 |
| CIITA | 1.37 | 0.64209 | 6.35 |
| RFX5 | 1 | 0.589277 | 1.47 |
| HLA-DRA | 1.32 | 0.101844 | 74.97 |
| HLA-DPA1 | 1.16 | 0.017043 | 4.79 |
| HLA-DPB1 | 1.04 | 0.679317 | 3.06 |
| CD74 | -1.14 | 0.107817 | 20.84 |
| HLA-DMA | 1.05 | 0.981515 | 2.98 |
| HLA-DMB | 1 | 0.974791 | 2.3 |
| CD86 | -1.04 | 0.308976 | 1.5 |
| CD40 | 2.03 | 0.001109 | 11.95 |
| PDCD1LG2 | 1.13 | 0.006731 | 11.63 |
| CD274 | 22.91 | 2.66E-08 | 35.96 |
| CTSB | -1.05 | 0.279003 | 1.49 |
| CTSL1 | 1.1 | 0.140328 | 1.47 |
| CTSS | 1.42 | 0.000001 | 2.97 |
| CTSO | 1.58 | 0.003884 | 9.25 |
| CTSZ | 1.3 | 0.002436 | 1.78 |
| CTSA | 1.23 | 0.000019 | 1.64 |
| B2M | 1.24 | 0.001358 | 1.54 |
| HLA-A | 1.17 | 0.005955 | 2.36 |
| HLA-B | 1.51 | 0.189464 | 2.55 |
| HLA-C | 1.14 | 0.011834 | 2.01 |
| HLA-E | 1.61 | 0.000029 | 2.73 |
| HLA-F | 1.18 | 0.016311 | 1.62 |
| HLA-G | 1.14 | 0.003628 | 2.21 |
| TAP1 | 12.55 | 4.12E-09 | 20.86 |
| TAP2 | 5.31 | 2.12E-08 | 11.88 |
| TAPBP | 1.42 | 0.000832 | 2.59 |
| PDIA3 | 1.79 | 0.006445 | 1.75 |
| CALR | 1.02 | 0.957583 | -1.24 |
